# Supplementary material for: 2D Boron Nanoplatelets as a Multifunctional Additive for Osteogenic, Gram‐Negative Antimicrobial and Mechanically Reinforcing Bone Repair Scaffolds
Source: Small Sci. 2025 Nov 4;5(12):e202500409. doi: 10.1002/smsc.202500409 (PMC12697802; doi:10.1002/smsc.202500409)
Supplement: Supplementary file 1 — Supplementary Material [file SMSC-5-e202500409-s001.pdf]

1 Supporting Information

2D Boron Nanoplatelets as a Multifunctional Additive for Osteogenic, Gram-Negative Anti-Microbial and Mechanically Reinforcing Bone Repair Scaffolds

*Jack Maughan, Harneet Kaur, Lucy Prendeville, Tian Carey, Cian O'Connor, Kevin Synnatschke, Juan Carlos Palomeque, Ian Woods, Fergal J. O'Brien, Jonathan N. Coleman**

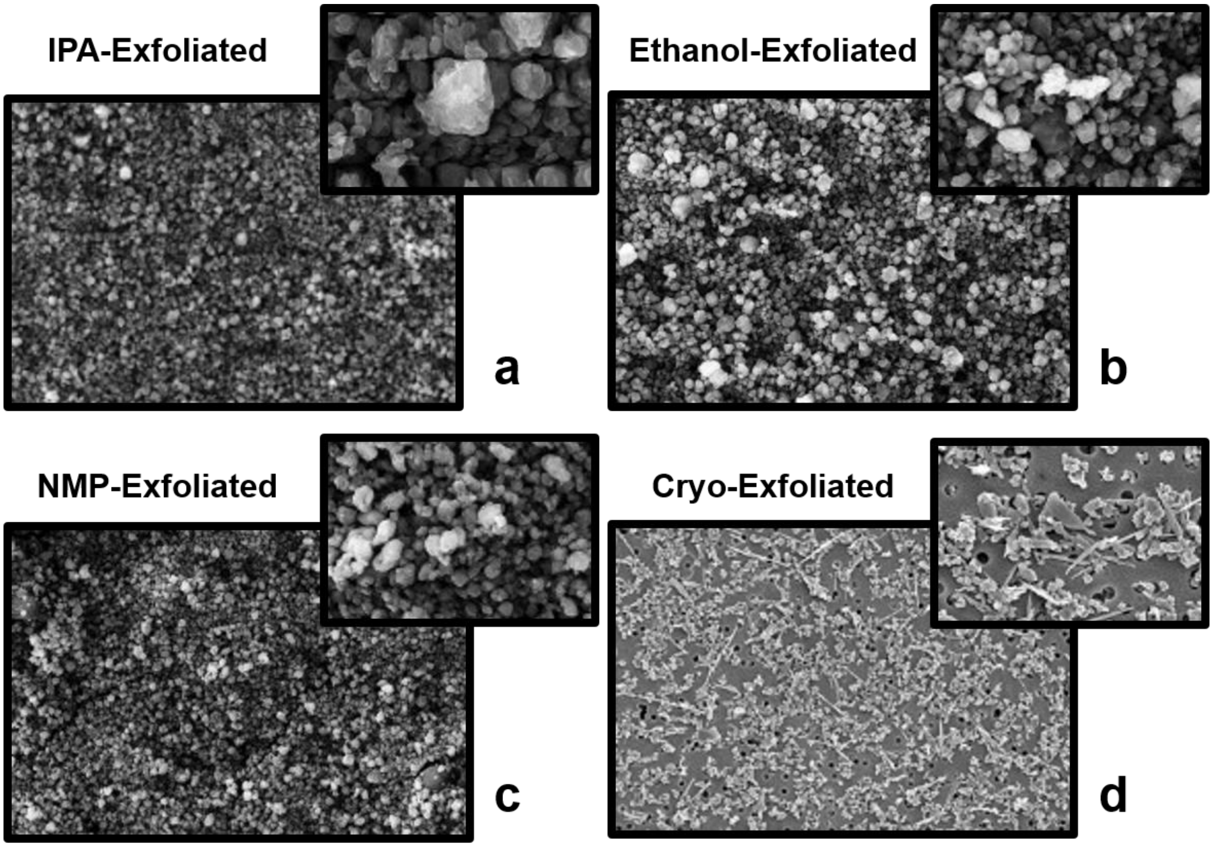

Fig. S1 Exfoliation of boron in various solvents: a-c) *Exfoliation of amorphous boron using a sonic tip in isopropyl alcohol (a), ethanol (b) and NMP (c), leading to nanoparticulate morphology.* ***d)*** *Cryogenically assisted exfoliation of amorphous boron in NMP, leading to a mix of 0D, 1D and 2D morphologies*

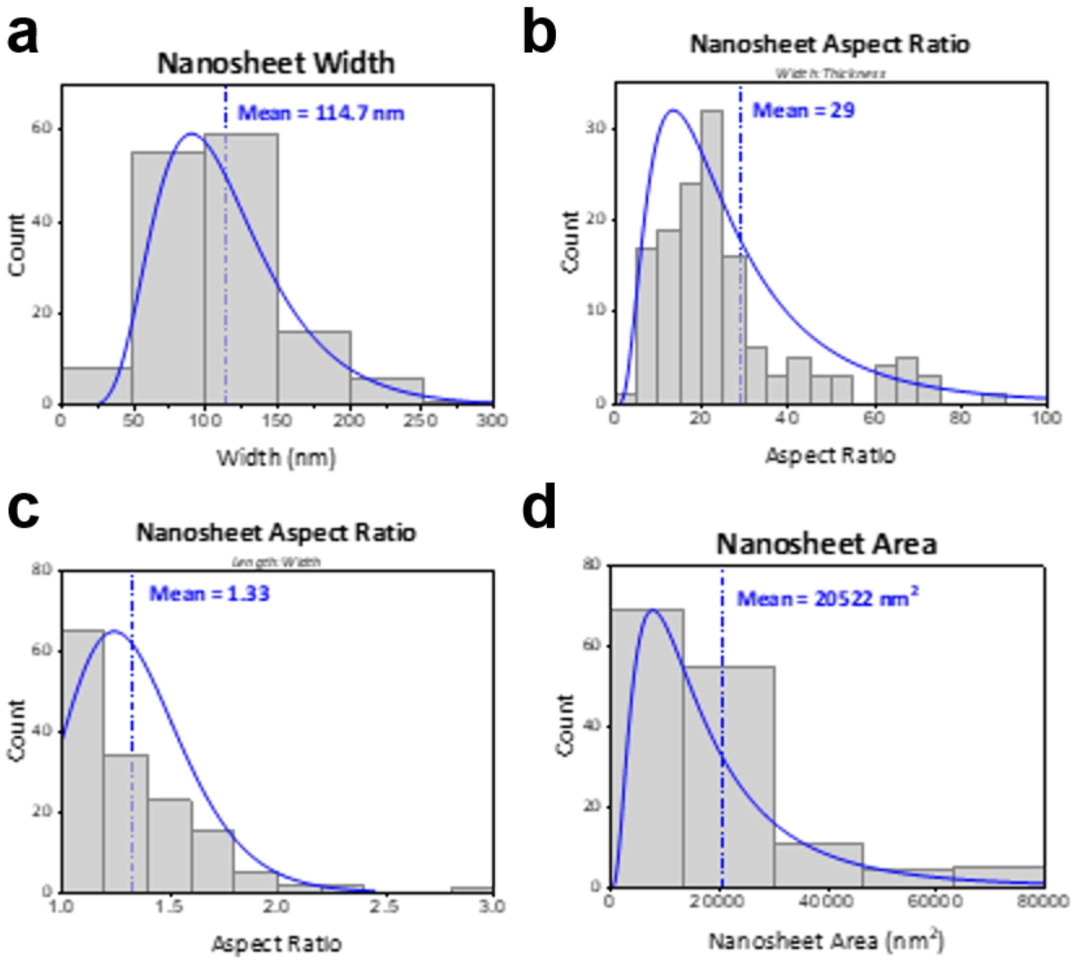

Fig. S2 Further AFM characterisation of boron nanoplatelets: a) *Nanosheet width* ***b)*** *Nanosheet aspect ratio (width:thickness)* ***c)*** *Nanosheet aspect ratio (length:width)* ***d)*** *Nanosheet area*

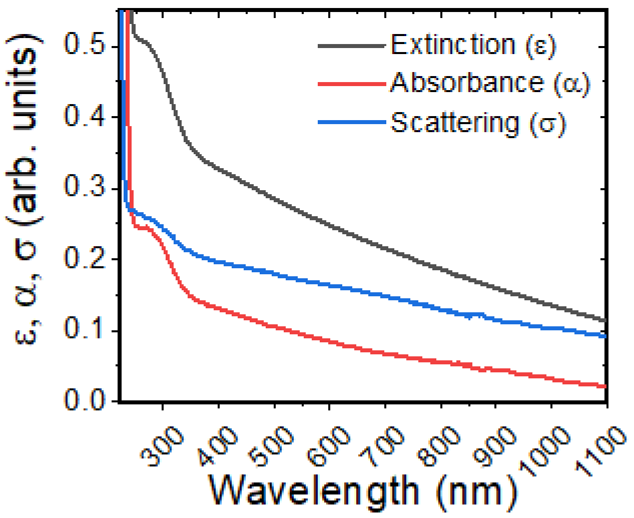

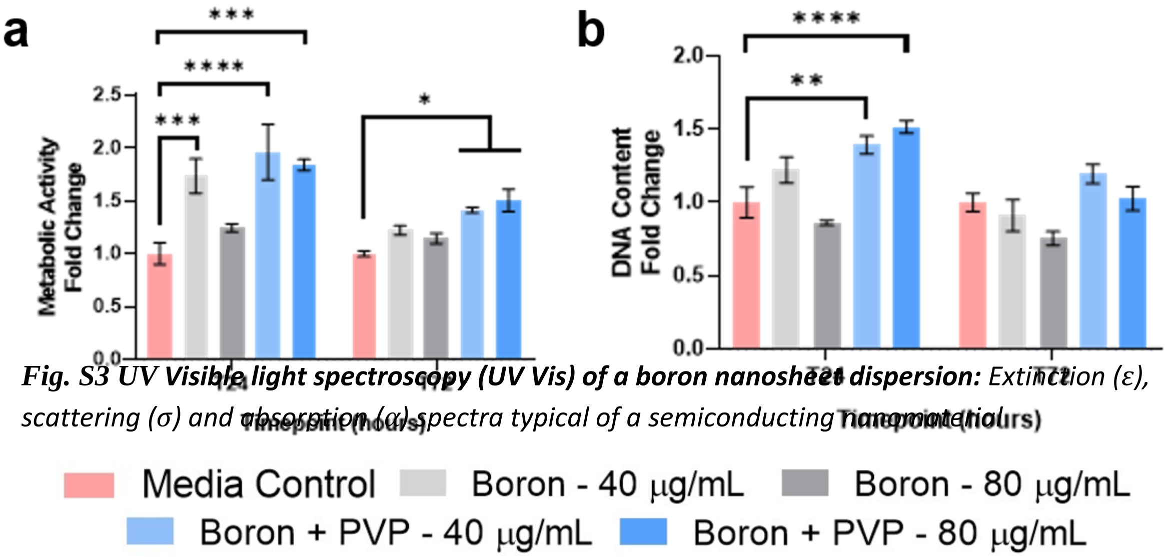

Fig. S3 ***UV Visible light spectroscopy (UV Vis) of a boron nanosheet dispersion:*** *Extinction (ε), scattering (σ) and absorption (α) spectra typical of a semiconducting nanomaterial.*

Fig. S4 Biocompatibility of boron stabilization: a-b) *It was necessary to determine if the nanosheets required any stabilization to remain dispersed and sufficiently hydrophilic in physiological conditions, as is the case with many nanomaterials of non-biological origin. To investigate this, mouse motor neuron cells (NSC-34 cell line) were grown in the presence of boron, with and without stabilization by PVP. This experiment demonstrated that PVP increased the biocompatibility of the boron nanosheets significantly, as seen by a significant increase in metabolic activity (a) and DNA content (b) for the PVP-stabilized material. Significances:* *$*p < 0.05$, $**p < 0.01$, $***p < 0.001$, $****p < 0.0001$*

Nanoplatelet

$$\text{Volume} = l \times w \times t$$

$$\text{Area} = 2 \times l \times w$$

$$\text{Area:Volume} = \frac{2 \times \cancel{l \times w}}{\cancel{l \times w} \times t} = \frac{2}{t}$$

Nanoparticle

$$\text{Volume} = \frac{4}{3}\pi r^3$$

$$\text{Area} = 4\pi r^2$$

$$\text{Area:Volume} = \frac{\cancel{4}\,\cancel{\pi r^2}}{\frac{\cancel{4}}{3}\,\cancel{\pi}\, r^{\cancel{3}}} = \frac{3}{r}$$

Surface Area to Volume Ratio

$$\therefore \text{Area:Volume}_{\text{nanoplatelet}} > \text{Area:Volume}_{\text{nanoparticle}}$$

$$\text{when } \frac{2}{t} > \frac{3}{r}$$

$$2r > 3t$$

$$t < \frac{2r}{3}$$

Fig. S5 Derivation for the higher surface area:volume ratio of a nanoplatelet compared with a spherical nanoparticle*: So long as the radius of the nanoparticle is >1.5 times the thickness of the nanoplatelet, the nanoplatelet will have a higher surface area:volume ratio*

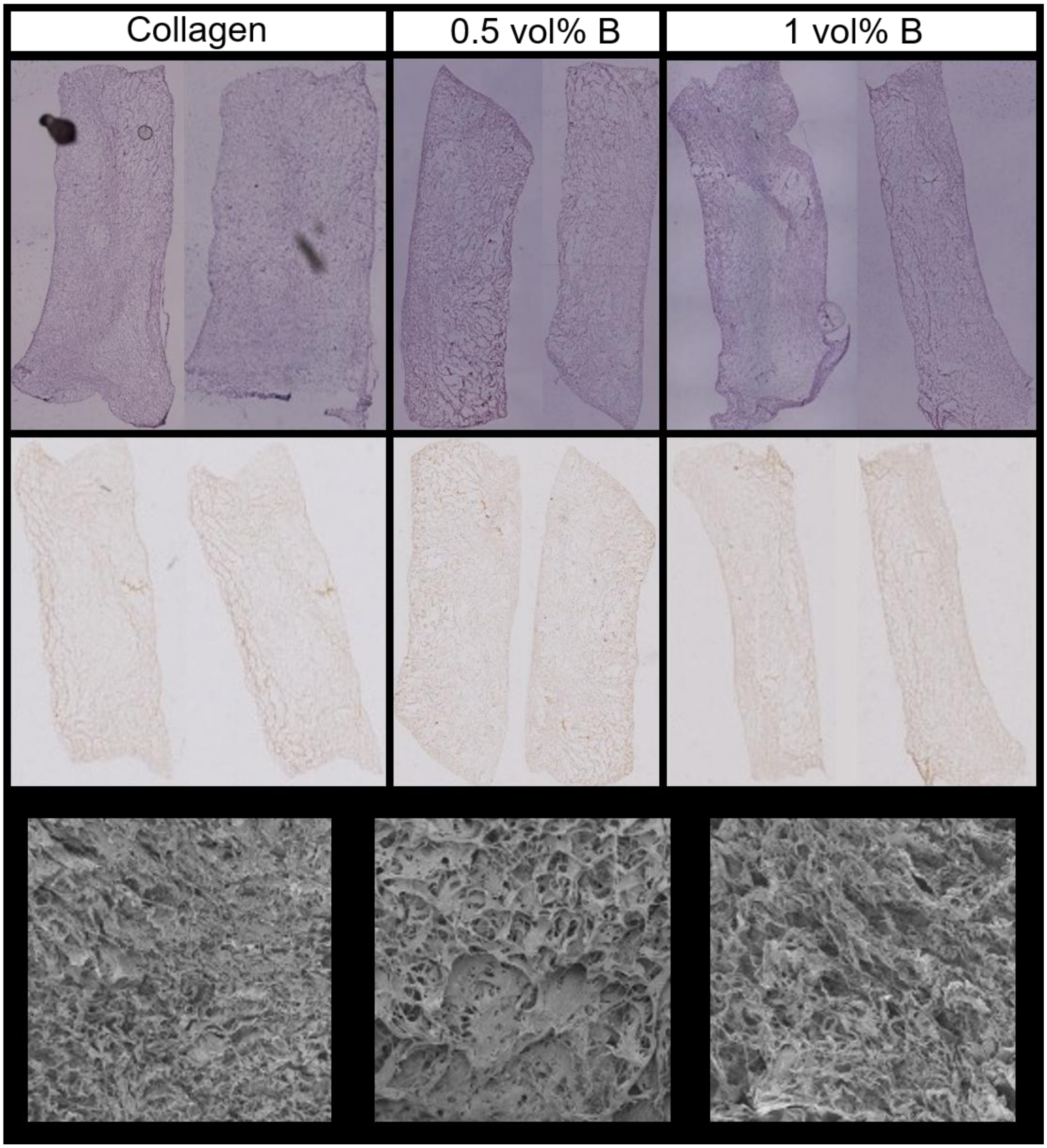

***Fig. S6 Histological staining of MC3T3s on BColl scaffolds: a)** H&E histological staining of collagen-boron scaffolds with MC3T3s. **b)** Alizarin Red histological staining of collagen-boron scaffolds with MC3T3s. Scalebars for A & B - 2 mm **c)** SEM of MC3T3s in collagen-boron scaffolds. Scalebars 100 µm*

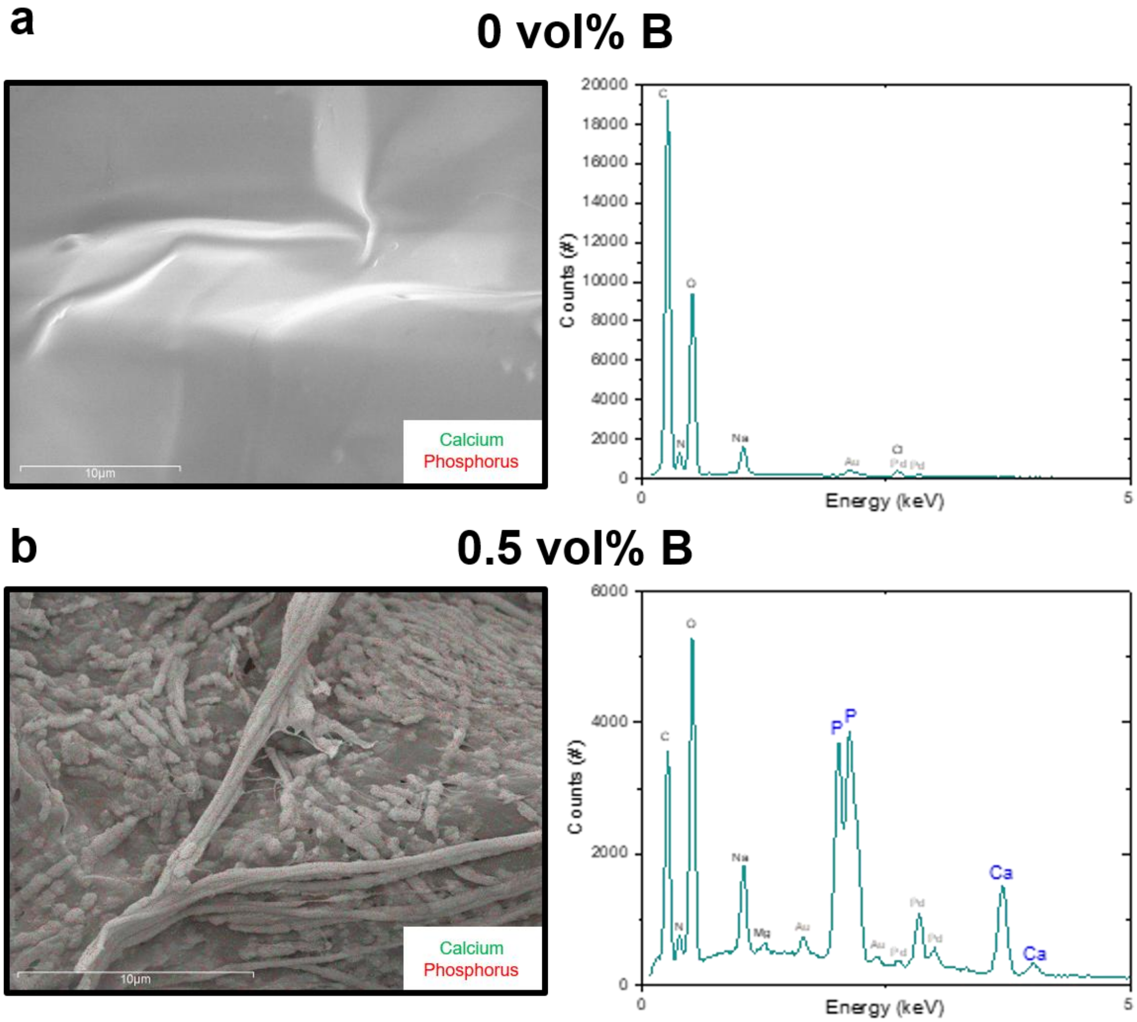

Fig. S7* *EDX of cell-laden BColl scaffolds: a-b) *EDX analysis of collagen (a) and BColl (b) scaffolds, showing that the deposits on boron-containing scaffolds consist of calcium phosphate, the main component of bone mineral, corroborating enhanced bone mineralization in the presence of boron*

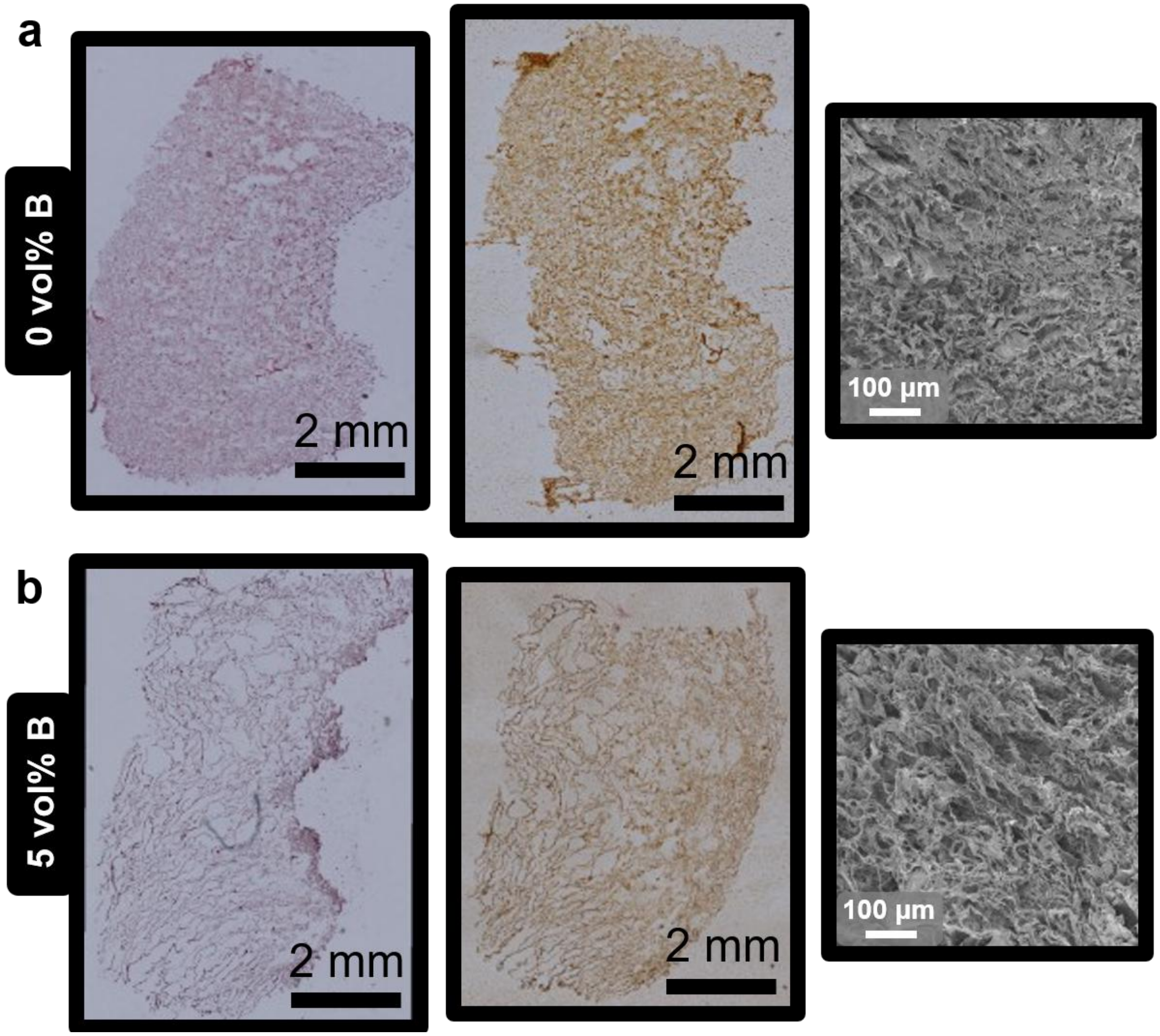

Fig. S8 Histological analysis of rat mesenchymal stem cells on BColl scaffolds: a-b) *To assess the extent and distribution of calcium deposition on the scaffolds, histological staining was carried out with H&E and Alizarin Red. Alizarin Red staining indicated robust mineralization of both collagen and BColl samples, throughout the sample. Scalebars 2 mm. SEM imaging was also carried out, to determine if there were any changes to the pore structure of the scaffolds following boron loading. Scalebars 100 µm*

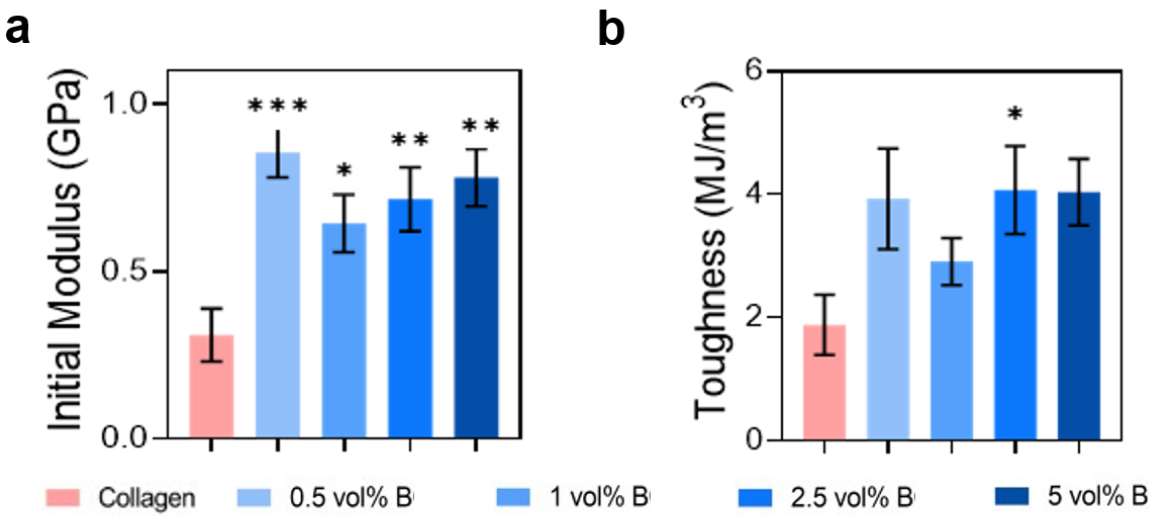

Fig. S9 Further mechanical analysis of BColl films: a) *Tensile modulus of BColl films in first 0.5% of strain, the regime most often experienced by cells, showing significant reinforcement in most BColl groups.* ***b)*** *Toughness measurements for BColl films, showing a trend towards increased toughness for all samples. Significances: *p < 0.05, **p < 0.01, ***p < 0.001, ****p < 0.0001*

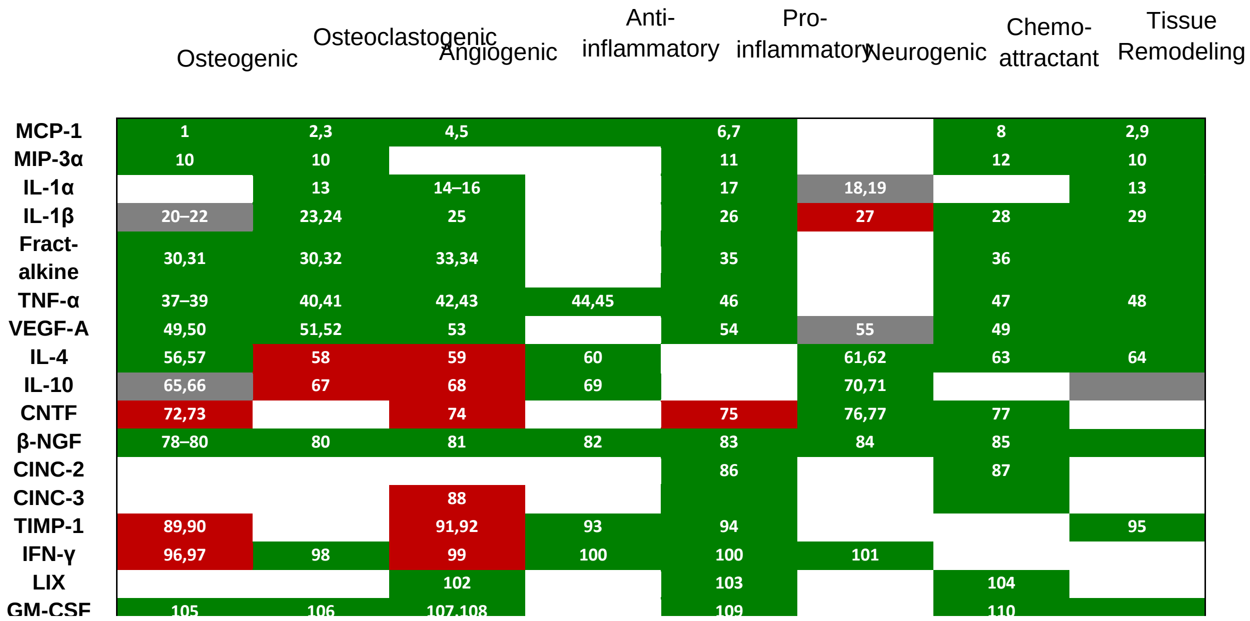

Fig. S10 Investigation of paracrine release profile of rMSCs on BColl scaffolds: *Matrix detailing the primary effects of each cytokine assessed in the ELISA data. Green corresponds to literature evidence of an effect, grey to contradictory or highly environment-dependent effect, red corresponds to evidence of a negative effect and white corresponds to no evidence for an effect. Numbers are citations*

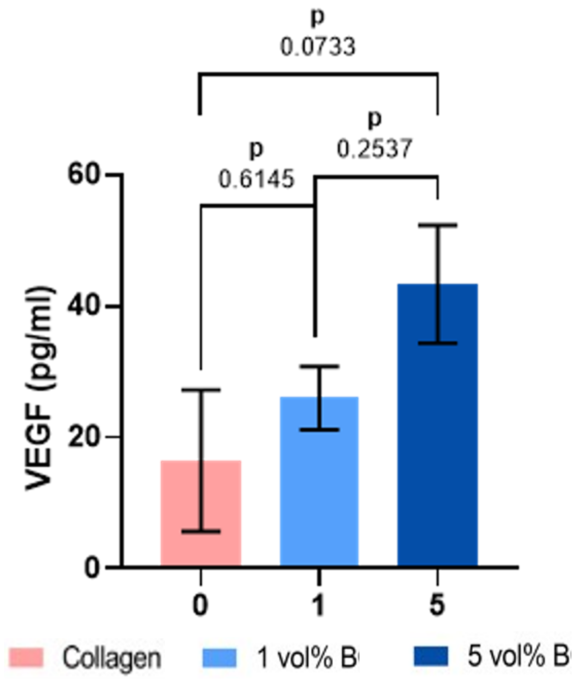

Fig. S11 Assessment of angiogenic effect of boron: *Analysis of VEGF release by human ELISA in rat MSCs after 3D scaffold culture for 28 days, showing trend towards increased VEGF release with boron addition*

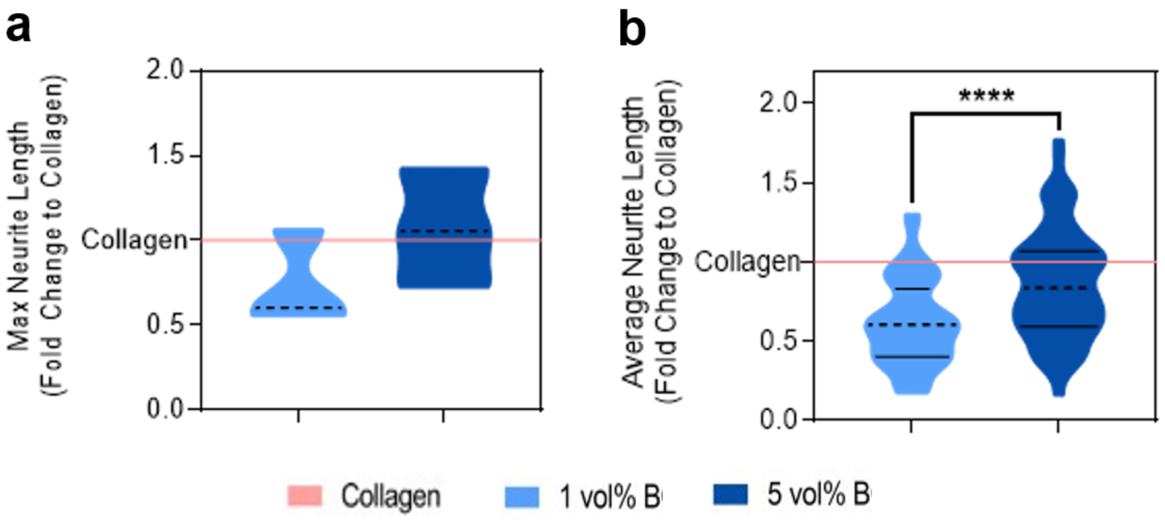

Fig. S12 Assessment of neurogenic effect of boron: a) *Max neurite length and* ***b)*** *average neurite length of DRGs grown on surface of BColl scaffolds for 14 days*

4.1 Supporting References

[1] M. Toya, N. Zhang, M. Tsubosaka, J. Kushioka, Q. Gao, X. Li, S. K.-H. Chow, S. B. Goodman, *Front. Cell Dev. Biol.* **2023**, *11*.

[2] B. S. Mulholland, M. R. Forwood, N. A. Morrison, *Curr Osteoporos Rep* **2019**, *17*, 538.

[3] J. A. Siddiqui, C. Le Henaff, J. Johnson, Z. He, D. B. Rifkin, N. C. Partridge, *Bone* **2021**, *143*, 115762.

[4] K. H. Hong, J. Ryu, K. H. Han, *Blood* **2005**, *105*, 1405.

[5] J. Niu, A. Azfer, O. Zhelyabovska, S. Fatma, P. E. Kolattukudy, *J Biol Chem* **2008**, *283*, 14542.

[6] T. L. Cranford, R. T. Enos, K. T. Velázquez, J. L. McClellan, J. M. Davis, U. P. Singh, M. Nagarkatti, P. S. Nagarkatti, C. M. Robinson, E. A. Murphy, *Int J Obes (Lond)* **2016**, *40*, 844.

[7] L. C. Becker, *Circulation Research* **2005**, *96*, 812.

[8] S. L. Deshmane, S. Kremlev, S. Amini, B. E. Sawaya, *J Interferon Cytokine Res* **2009**, *29*, 313.

[9] H. J. Jürgensen, L. M. Silva, O. Krigslund, S. van Putten, D. H. Madsen, N. Behrendt, L. H. Engelholm, T. H. Bugge, *Matrix Biol Plus* **2019**, *1*, 100003.

[10] G. Lisignoli, A. Piacentini, S. Cristino, F. Grassi, C. Cavallo, L. Cattini, B. Tonnarelli, C. Manferdini, A. Facchini, *Journal Cellular Physiology* **2007**, *210*, 798.

[11] A. Izadpanah, M. B. Dwinell, L. Eckmann, N. M. Varki, M. F. Kagnoff, *Am J Physiol Gastrointest Liver Physiol* **2001**, *280*, G710.

[12] N. Aziz, R. Detels, L. C. Chang, A. W. Butch, *J AIDS Clin Res* **2016**, *7*, 587.

[13] Y.-M. Lee, N. Fujikado, H. Manaka, H. Yasuda, Y. Iwakura, *International Immunology* **2010**, *22*, 805.

[14] P. Salven, K. Hattori, B. Heissig, S. Rafii, *FASEB J* **2002**, *16*, 1471.

[15] A. Naldini, F. Carraro, *CDTIA* **2005**, *4*, 3.

[16] E. Voronov, Y. Carmi, R. N. Apte, *Front. Physiol.* **2014**, *5*.

[17] A. Malik, T.-D. Kanneganti, *Immunol Rev* **2018**, *281*, 124.

[18] K. E. Salmeron, M. E. Maniskas, D. N. Edwards, R. Wong, I. Rajkovic, A. Trout, A. A. Rahman, S. Hamilton, J. F. Fraser, E. Pinteaux, G. J. Bix, *Journal of Neuroinflammation* **2019**, *16*, 222.

[19] K. N. Murray, A. R. Parry-Jones, S. M. Allan, *Front Cell Neurosci* **2015**, *9*, 18.

[20] H. Wang, Z. Ni, J. Yang, M. Li, L. Liu, X. Pan, L. Xu, X. Wang, S. Fang, *Exp Ther Med* **2020**, *20*, 3001.

[21] D. C. Lacey, P. J. Simmons, S. E. Graves, J. A. Hamilton, *Osteoarthritis and Cartilage* **2009**, *17*, 735.

[22] C. Mao, Y. Wang, X. Zhang, X. Zheng, T. Tang, E. Lu, *Cell Death Dis* **2016**, *7*, e2296.

[23] J. H. Kim, H. M. Jin, K. Kim, I. Song, B. U. Youn, K. Matsuo, N. Kim, *J Immunol* **2009**, *183*, 1862.

[24] Y. Otsuka, T. Kondo, H. Aoki, Y. Goto, Y. Kawaguchi, Y. Waguri-Nagaya, K. Miyazawa, S. Goto, M. Aoyama, *Journal of Pharmacological Sciences* **2023**, *151*, 1.

[25] E. Fahey, S. L. Doyle, *Frontiers in Immunology* **2019**, *10*.

[26] K. Ren, R. Torres, *Brain Res Rev* **2009**, *60*, 57.

[27] J. W. Koo, R. S. Duman, *Proc Natl Acad Sci U S A* **2008**, *105*, 751.

[28] G. W. Hunninghake, A. J. Glazier, M. M. Monick, C. A. Dinarello, *Am Rev Respir Dis* **1987**, *135*, 66.

[29] R. A. Dodds, K. Merry, A. Littlewood, M. Gowen, *J Histochem Cytochem.* **1994**, *42*, 733.

[30] K. Koizumi, Y. Saitoh, T. Minami, N. Takeno, K. Tsuneyama, T. Miyahara, T. Nakayama, H. Sakurai, Y. Takano, M. Nishimura, T. Imai, O. Yoshie, I. Saiki, *The Journal of Immunology* **2009**, *183*, 7825.

[31] T. Yang, L. Guo, L. Chen, J. Li, Q. Li, Y. Pi, J. Zhu, L. Zhang, *Cell Calcium* **2020**, *91*, 102265.

[32] Y. Kuboi, Y. Kuroda, M. Ohkuro, S. Motoi, Y. Tomimori, H. Yasuda, N. Yasuda, T. Imai, K. Matsuo, *JBMR Plus* **2022**, *6*, e10680.

[33] J. A. Kim, J.-Y. Kwak, Y. Eunjung, J. Lee, Y. Park, H. E. Broxmeyer, *Blood* **2016**, *128*, 2507.

[34] M. V. Volin, J. M. Woods, M. A. Amin, M. A. Connors, L. A. Harlow, A. E. Koch, *Am J Pathol* **2001**, *159*, 1521.

[35] B. A. Jones, M. Beamer, S. Ahmed, *Mol Interv* **2010**, *10*, 263.

[36] P. Wojdasiewicz, Ł. A. Poniatowski, A. Kotela, J. Deszczyński, I. Kotela, D. Szukiewicz, *Arch Immunol Ther Exp (Warsz)* **2014**, *62*, 395.

[37] S. Daniele, L. Natali, C. Giacomelli, P. Campiglia, E. Novellino, C. Martini, M. L. Trincavelli, *Mol Cell Biol* **2017**, *37*, e00442.

[38] Q. Mo, W. Zhang, A. Zhu, L. J. Backman, J. Chen, *Human Cell* **2022**, *35*, 957.

[39] K. Hess, A. Ushmorov, J. Fiedler, R. E. Brenner, T. Wirth, *Bone* **2009**, *45*, 367.

[40] J. Lam, S. Takeshita, J. E. Barker, O. Kanagawa, F. P. Ross, S. L. Teitelbaum, *J Clin Invest* **2000**, *106*, 1481.

[41] G. Luo, F. Li, X. Li, Z.-G. Wang, B. Zhang, *Mol Med Rep* **2018**, *17*, 6605.

[42] L. F. Fajardo, H. H. Kwan, J. Kowalski, A. C. Allisont, *The American Journal of Pathology* **1992**, *140*.

[43] S. J. Leibovich, P. J. Polverini, H. M. Shepard, D. M. Wiseman, V. Shively, N. Nuseir, *Nature* **1987**, *329*, 630.

[44] M. Zakharova, H. K. Ziegler, *The Journal of Immunology* **2005**, *175*, 5024.

[45] S. Masli, B. Turpie, *Immunology* **2009**, *127*, 62.

[46] G. van Loo, M. J. M. Bertrand, *Nat Rev Immunol* **2023**, *23*, 289.

[47] Y. Torrente, E. E. Fahime, N. J. Caron, R. Del Bo, M. Belicchi, F. Pisati, J. P. Tremblay, N. Bresolin, *Cell Transplant* **2003**, *12*, 91.

[48] G. E. Glass, J. K. Chan, A. Freidin, M. Feldmann, N. J. Horwood, J. Nanchahal, *Proc Natl Acad Sci U S A* **2011**, *108*, 1585.

[49] U. Mayr-Wohlfart, J. Waltenberger, H. Hausser, S. Kessler, K.-P. Günther, C. Dehio, W. Puhl, R. E. Brenner, *Bone* **2002**, *30*, 472.

[50] K. Hu, B. R. Olsen, *Bone* **2016**, *91*, 30.

[51] S. E. Aldridge, T. W. J. Lennard, J. R. Williams, M. A. Birch, *Biochem Biophys Res Commun* **2005**, *335*, 793.

[52] H.-R. Kim, K.-W. Kim, B.-M. Kim, M.-L. Cho, S.-H. Lee, *PLoS One* **2015**, *10*, e0124909.

[53] H. M. W. Verheul, H. M. Pinedo, *Clinical Breast Cancer* **2000**, *1*, S80.

[54] M. E. J. Reinders, M. Sho, A. Izawa, P. Wang, D. Mukhopadhyay, K. E. Koss, C. S. Geehan, A. D. Luster, M. H. Sayegh, D. M. Briscoe, *J Clin Invest* **2003**, *112*, 1655.

[55] S.-W. Shen, C.-L. Duan, X.-H. Chen, Y.-Q. Wang, X. Sun, Q.-W. Zhang, H.-R. Cui, F.-Y. Sun, *Neuropharmacology* **2016**, *108*, 451.

[56] D. Li, X. Li, J. Zhang, Z. Tang, A. Tian, *Front. Immunol.* **2023**, *14*.

[57] J. Zhang, H. Shi, N. Zhang, L. Hu, W. Jing, J. Pan, *Cell Prolif* **2020**, *53*, e12907.

[58] A. Yamada, M. Takami, T. Kawawa, R. Yasuhara, B. Zhao, A. Mochizuki, Y. Miyamoto, T. Eto, H. Yasuda, Y. Nakamichi, N. Kim, T. Katagiri, T. Suda, R. Kamijo, *Immunology* **2007**, *120*, 573.

[59] O. V. Volpert, T. Fong, A. E. Koch, J. D. Peterson, C. Waltenbaugh, R. I. Tepper, N. P. Bouck, *J Exp Med* **1998**, *188*, 1039.

[60] E. A. Woodward, C. M. Prêle, S. E. Nicholson, T. B. Kolesnik, P. H. Hart, *Immunology* **2010**, *131*, 118.

[61] D. Pan, L. Schellhardt, J. A. Acevedo-Cintron, D. Hunter, A. K. Snyder-Warwick, S. E. Mackinnon, M. D. Wood, *Experimental Neurology* **2022**, *347*, 113909.

[62] J. M. Daines, L. Schellhardt, M. D. Wood, *Neurorehabil Neural Repair* **2021**, *35*, 431.

[63] M. T. A. Villar, J. A. Douglass, C. Heusser, P. Bradding, S. T. Holgate, M. K. Church, In *The Chemokines: Biology of the Inflammatory Peptide Supergene Family II* (Eds.: Lindley, I. J. D.; Westwick, J.; Kunkel, S.), Springer US, Boston, MA, **1993**, pp. 191–191.

[64] L. Bosurgi, Y. G. Cao, M. Cabeza-Cabrerizo, A. Tucci, L. D. Hughes, Y. Kong, J. S. Weinstein, P. Licona-Limon, E. T. Schmid, F. Pelorosso, N. Gagliani, J. E. Craft, R. A. Flavell, S. Ghosh, C. V. Rothlin, *Science* **2017**, *356*, 1072.

[65] L. Yuan, H. You, N. Qin, W. Zuo, *Cellular Reprogramming* **2021**, *23*, 270.

[66] P. Van Vlasselaer, B. Borremans, R. Van Den Heuvel, U. Van Gorp, R. de Waal Malefyt, *Blood* **1993**, *82*, 2361.

[67] K. E. Evans, S. W. Fox, *BMC Cell Biol* **2007**, *8*, 4.

[68] D. S. Dace, A. A. Khan, J. Kelly, R. S. Apte, *PLoS One* **2008**, *3*, e3381.

[69] E. H. Steen, X. Wang, S. Balaji, M. J. Butte, P. L. Bollyky, S. G. Keswani, *Adv Wound Care (New Rochelle)* **2020**, *9*, 184.

[70] C. Patilas, I. Varsamos, A. Galanis, M. Vavourakis, D. Zachariou, V. Marougklianis, I. Kolovos, G. Tsalimas, P. Karampinas, A. Kaspiris, J. Vlamis, S. Pneumaticos, *Diagnostics* **2024**, *14*, 151.

[71] J. Li, P. Wang, T. Zhou, W. Jiang, H. Wu, S. Zhang, L. Deng, H. Wang, *Front. Mol. Neurosci.* **2023**, *16*.

[72] N. E. McGregor, I. J. Poulton, E. C. Walker, S. Pompolo, J. M. W. Quinn, T. J. Martin, N. A. Sims, *Calcif Tissue Int* **2010**, *86*, 261.

[73] J. Yong, S. Gröger, J. von Bremen, S. Ruf, *Int J Mol Sci* **2022**, *23*, 9311.

[74] F. Bucher, E. Aguilar, K. V. Marra, J. Rapp, J. Arnold, S. Diaz-Aguilar, C. Lange, H. Agostini, G. Schlunck, A. Stahl, M. Friedlander, *Invest Ophthalmol Vis Sci* **2020**, *61*, 20.

[75] H.-W. Lin, M. R. Jain, H. Li, S. W. Levison, *J Neuroinflammation* **2009**, *6*, 7.

[76] E. Rockenstein, K. Ubhi, E. Doppler, P. Novak, H. Moessler, B. Li, J. Blanchard, I. Grundke-Iqbal, K. Iqbal, M. Mante, A. Adame, L. Crews, E. Masliah, *J Alzheimers Dis* **2011**, *27*, 743.

[77] L.-P. Cen, J.-M. Luo, C.-W. Zhang, Y.-M. Fan, Y. Song, K.-F. So, N. van Rooijen, C. P. Pang, D. S. C. Lam, Q. Cui, *Invest Ophthalmol Vis Sci* **2007**, *48*, 4257.

[78] K. O. Rivera, F. Russo, R. M. Boileau, R. E. Tomlinson, T. Miclau, R. S. Marcucio, T. A. Desai, C. S. Bahney, *Sci Rep* **2020**, *10*, 22241.

[79] S. Yang, J. Cheng, C. Man, L. Jiang, G. Long, W. Zhao, D. Zheng, *Journal of Orthopaedic Surgery and Research* **2021**, *16*, 74.

[80] F. Hemingway, R. Taylor, H. J. Knowles, N. A. Athanasou, *Bone* **2011**, *48*, 938.

[81] W.-H. Chen, C.-Q. Mao, L.-L. Zhuo, J. L. Ong, *Neural Regen Res* **2015**, *10*, 1159.

[82] G. Prencipe, G. Minnone, R. Strippoli, L. De Pasquale, S. Petrini, I. Caiello, L. Manni, F. De Benedetti, L. Bracci-Laudiero, *The Journal of Immunology* **2014**, *192*, 3345.

[83] G. Minnone, F. De Benedetti, L. Bracci-Laudiero, *Int J Mol Sci* **2017**, *18*, 1028.

[84] S. D. Skaper, *Immunology* **2017**, *151*, 1.

[85] J. Sawada, A. Itakura, A. Tanaka, T. Furusaka, H. Matsuda, *Blood* **2000**, *95*, 2052.

[86] H. Nakagawa, S. Shiota, K. Takano, F. Shibata, H. Kato, *Biochemical and Biophysical Research Communications* **1996**, *220*, 945.

[87] K. Takano, H. Nakagawa, *Inflamm Res* **2001**, *50*, 503.

[88] J. Jenkins, E. Wagner, *J Vis Exp* **2013**, 50217.

[89] T. Liang, W. Gao, L. Zhu, J. Ren, H. Yao, K. Wang, D. Shi, *Biosci Rep* **2019**, *39*, BSR20181290.

[90] V. Egea, S. Zahler, N. Rieth, P. Neth, T. Popp, K. Kehe, M. Jochum, C. Ries, *Proceedings of the National Academy of Sciences* **2012**, *109*, E309.

[91] M. J. Reed, T. Koike, E. Sadoun, E. H. Sage, P. Puolakkainen, *Microvasc Res* **2003**, *65*, 9.

[92] Q. X. A. Sang, *Cell Res* **1998**, *8*, 171.

[93] E.-J. Lee, H.-S. Kim, *J Neuroinflammation* **2014**, *11*, 116.

[94] B. Schoeps, J. Frädrich, A. Krüger, *Trends in Cell Biology* **2023**, *33*, 413.

[95] G. A. Cabral-Pacheco, I. Garza-Veloz, C. Castruita-De la Rosa, J. M. Ramirez-Acuña, B. A. Perez-Romero, J. F. Guerrero-Rodriguez, N. Martinez-Avila, M. L. Martinez-Fierro, *Int J Mol Sci* **2020**, *21*, 9739.

[96] J.-H. Lai, Y.-P. Hsu, C.-H. Yang, Y.-H. Chen, C.-C. Liu, S.-K. Chen, *Mol Biol Rep* **2022**, *49*, 12007.

[97] A. S. Dighe, S. Yang, V. Madhu, G. Balian, Q. Cui, *J Orthop Res* **2013**, *31*, 227.

[98] Y. Gao, F. Grassi, M. R. Ryan, M. Terauchi, K. Page, X. Yang, M. N. Weitzmann, R. Pacifici, *J Clin Invest* **2007**, *117*, 122.

[99] N. Sato, H. Nariuchi, N. Tsuruoka, T. Nishihara, J. G. Beitz, P. Calabresi, A. R. Frackelton, *J Invest Dermatol* **1990**, *95*, 85S.

[100] H. Mühl, J. Pfeilschifter, *Int Immunopharmacol* **2003**, *3*, 1247.

[101] X. Yuan, F. He, F. Zheng, Y. Xu, J. Zou, *Neuroscience* **2020**, *448*, 219.

[102] A. Moldobaeva, A. Baek, L. Eldridge, E. M. Wagner, *Microvascular Research* **2010**, *80*, 18.

[103] B. Chandrasekar, P. C. Melby, H. M. Sarau, M. Raveendran, R. P. Perla, F. M. Marelli-Berg, N. O. Dulin, I. S. Singh, *J Biol Chem* **2003**, *278*, 4675.

[104] L. E. Rovai, H. R. Herschman, J. B. Smith, *J Leukoc Biol* **1998**, *64*, 494.

[105] F. Roseren, M. Pithioux, S. Robert, L. Balasse, B. Guillet, E. Lamy, S. Roffino, *Int J Mol Sci* **2021**, *22*, 3505.

[106] K. Nomura, S. Kuroda, H. Yoshikawa, T. Tomita, *Biochemical and Biophysical Research Communications* **2008**, *367*, 881.

[107] J. Zhao, L. Chen, B. Shu, J. Tang, L. Zhang, J. Xie, S. Qi, Y. Xu, *PLoS One* **2014**, *9*, e92691.

[108] S. Wallner, S. Peters, C. Pitzer, H. Resch, U. Bogdahn, A. Schneider, *Front. Cell Dev. Biol.* **2015**, *3*.

[109] P. Bhattacharya, I. Budnick, M. Singh, M. Thiruppathi, K. Alharshawi, H. Elshabrawy, M. J. Holterman, B. S. Prabhakar, *J Interferon Cytokine Res* **2015**, *35*, 585.

[110] M. Khajah, B. Millen, D. C. Cara, C. Waterhouse, D.-M. McCafferty, *J Leukoc Biol* **2011**, *89*, 945.

[111] Y.-T. Park, S.-M. Lee, X. Kou, B. Karabucak, *J Endod* **2019**, *45*, 1342.

[112] R.-L. Huang, Y. Sun, C.-K. Ho, K. Liu, Q.-Q. Tang, Y. Xie, Q. Li, *Cell Death Dis* **2018**, *9*, 1.

[113] N. E. McGregor, M. Murat, J. Elango, I. J. Poulton, E. C. Walker, B. Crimeen-Irwin, P. W. M. Ho, J. H. Gooi, T. J. Martin, N. A. Sims, *J Biol Chem* **2019**, *294*, 7850.

[114] N. Udagawa, N. Takahashi, T. Katagiri, T. Tamura, S. Wada, D. M. Findlay, T. J. Martin, H. Hirota, T. Taga, T. Kishimoto, T. Suda, *The Journal of experimental medicine* **1995**, *182*, 1461.

[115] T. Cohen, D. Nahari, L. W. Cerem, G. Neufeld, B.-Z. Levi, *Journal of Biological Chemistry* **1996**, *271*, 736.

[116] G. Gopinathan, C. Milagre, O. M. T. Pearce, L. E. Reynolds, K. Hodivala-Dilke, D. A. Leinster, H. Zhong, R. E. Hollingsworth, R. Thompson, J. R. Whiteford, F. Balkwill, *Cancer Res* **2015**, *75*, 3098.

[117] Z. Xing, J. Gauldie, G. Cox, H. Baumann, M. Jordana, X. F. Lei, M. K. Achong, *J Clin Invest* **1998**, *101*, 311.

[118] T. Tanaka, M. Narazaki, T. Kishimoto, *Cold Spring Harb Perspect Biol* **2014**, *6*, a016295.

[119] M. Pedroza, D. J. Schneider, H. Karmouty-Quintana, J. Coote, S. Shaw, R. Corrigan, J. G. Molina, J. L. Alcorn, D. Galas, R. Gelinas, M. R. Blackburn, *PLoS One* **2011**, *6*, e22667.

[120] K. Włodarski, P. Włodarski, *Ortop Traumatol Rehabil* **2009**, *11*, 1.

[121] P. Ducy, M. Amling, S. Takeda, M. Priemel, A. F. Schilling, F. T. Beil, J. Shen, C. Vinson, J. M. Rueger, G. Karsenty, *Cell* **2000**, *100*, 197.

[122] W. R. Holloway, F. M. Collier, C. J. Aitken, D. E. Myers, J. M. Hodge, M. Malakellis, T. J. Gough, G. R. Collier, G. C. Nicholson, *J Bone Miner Res* **2002**, *17*, 200.

[123] E. Bernotiene, G. Palmer, C. Gabay, *Arthritis Research & Therapy* **2006**, *8*, 217.

[124] G. Dessie, B. Ayelign, Y. Akalu, T. Shibabaw, M. D. Molla, *DMSO* **2021**, *14*, 3307.

[125] M. L. Calió, A. C. Mosini, D. S. Marinho, G. N. Salles, F. H. Massinhani, G. M. Ko, M. A. Porcionatto, *Neurobiology of Disease* **2021**, *148*, 105219.

[126] F. Montecucco, G. Bianchi, P. Gnerre, M. Bertolotto, F. Dallegri, L. Ottonello, *Ann N Y Acad Sci* **2006**, *1069*, 463.

[127] K. J. Motyl, C. J. Rosen, *Biochimie* **2012**, *94*, 2089.
